# Spiritual well-being correlates with quality of life of both cancer and non-cancer patients in palliative care - further validation of EORTC QLQ-SWB32 in Finnish

**DOI:** 10.1186/s12904-023-01153-0

**Published:** 2023-03-30

**Authors:** Raimo Goyarrola, Jari Lipsanen, Suvi-Maria Saarelainen, Raili Suviranta, Eeva Rahko, Annamarja Lamminmäki, Tuula Klaavuniemi, Satu Ahtiluoto, Antti Ohvanainen, Pekka Metso, Reino Pöyhiä

**Affiliations:** 1 School of Medicine, University of Eastern Finland, Kuopio, Finland; 2 Department of Statistics, University of Helsinki, Helsinki, Finland; 3 School of Theology, Philosophical Faculty, University of Eastern Finland, Joensuu, Finland; 4 Diaconia Journal, Evangelical Lutheran Church of Finland, Helsinki, Finland; 5 Department of Oncology, University Hospital, Oulu, Finland; 6 Department of Oncology, University Hospital, Kuopio, Finland; 7 Department of Oncology, Central Hospital, Mikkeli, Finland; 8 Helsinki University Hospital, Helsinki, Finland; 9 Palliative care unit and hospital at home, Siun sote, Joensuu, Finland; 10 Palliative Center, Essote, Mikkeli, Finland

**Keywords:** Palliative care, Spirituality, Quality of life, Surveys and questionnaires

## Abstract

**Background:**

The European Organisation for Research and Treatment of Cancer (EORTC) has developed the Spiritual Well-being Questionnaire (EORTC QLQ-SWB32), a measure of spiritual well-being validated with people receiving palliative care for cancer, although its usefulness is not restricted to that population. We aimed to translate and validate this tool in Finnish and to study the relationship between spiritual well-being (SWB) and quality of life (QOL).

**Methods:**

A Finnish translation was produced according to the guidelines of EORTC and included forward- and back-translations. Face, content, construct and convergence/divergence validity and reliability were studied in a prospective manner. QOL was assessed with EORTC QLQ-C30 and 15D questionnaires.

Sixteen individuals participated in the pilot testing. 101 cancer patients drawn from oncology units, and 89 patients with other chronic diseases drawn from religious communities in different parts of the country participated in the validation stage. Retest was obtained from 16 individuals (8 cancer and 8 non-cancer patients). Inclusion criteria included patients with either a well-defined palliative care plan, or who would benefit from palliative care, as well as the capacity to understand and communicate in Finnish.

**Results:**

The translation appeared understandable and acceptable. Factorial analysis identified four scoring scales with high Cronbach alfa values: Relationship with Self (0.73), Relationship with Others (0.84), Relationship with Something Greater (0.82), Existential (0.81), and, additionally, a scale on Relationship with God (0.85). There was a significant correlation between SWB and QOL in all participants.

**Conclusions:**

The Finnish translation of EORTC QLQ-SWB32 is a valid and reliable measure both for research and clinical practice. SWB is correlated with QOL in cancer and non-cancer patients undergoing palliative care or who are eligible for it.

## Background

The World Health Organization emphasizes the need to integrate the spiritual dimension of health and spirituality in palliative care as a means for improving the quality of life (QOL) [1]. Life-threatening or long-term disease indeed often activates questions about death and a life beyond mere biological or physical experience [2–4]. Especially when the approach of death is foreseen, the value of one´s own life, the meaning of suffering, relationships with other people and God, the need for reconciliation, forgiveness, and life after death become important questions [5–7]. Some studies suggest that these dimensions should be understood as spirituality [8–11].

In 2009 a Consensus Conference with the aim to improve the quality of spiritual care agreed on the definition of spirituality as that aspect of humanity that refers to the way individuals seek and express meaning and purpose and the way they experience their connectedness to the moment, to self, to others, to nature, and to the significant other or sacred [12].

It has been noted that there exist contextual differences between the U.S. and Europe [13], which impacts on the understanding of spirituality. Current studies conducted in the area of health and religion have shown that in a secularized European context, during a personal health crisis, three existential domains intersect: religious, spiritual and secular. We adhere to the definition of the religious existential domain as that based on a theistic faith which includes: a personal belief in God, personal meditation on the existence of God, and the practice of rituals and rites to reach this God. Spirituality on the other hand is seen as a more personal construct related to the ultimate meaning of life. At the same time, spirituality always bears a connection to a transcendent reality. Individuals who have not formed any religious or spiritual views or transcendent connections in life still encounter and ponder existential questions on meaning. The secular existential domain encompasses these types of experiences that are not linked to any transcendent reality [14–16].

Despite the variety of definitions, studies share the view that spirituality is an important factor in improving quality of life (QOL) [17–23], and even as a predictive construct of QOL [24, 25]. Palliative care is holistic care, which includes addressing the spirituality of the patient [26–30], and in this sense, spiritual assessment and intervention should be considered important in palliative care [13, 31, 32]. However, there continue to exist significant challenges in determining the indicators of spirituality in the care of patients [16, 33–35]. Several instruments have been developed for the assessment of spirituality in palliative care [36]. Measurements of spiritual well-being (SWB) have been used as indicators of an individual´s spirituality and its association with QOL [37].

Recent studies suggest that spirituality and SWB are two separate dimensions, although they tend to correlate with each other. SWB can be understood as the harmony that forms when the individual has acquired the adequate balance between the self, the significant other, nature and the transcendent /God [38].

A systematic review found a relationship between SWB and QOL in cancer patients [39]. This relationship has also been shown to exist for palliative-care patients [40]. Another recent review described 152 tools to explore SWB, but only a few of them have been properly validated [41]. Among these tools, the Spiritual Well-being questionnaire, EORTC QLQ-SWB32 (SWB32) of The European Organisation for Research and Treatment of Cancer Quality of Life Group (EORTC) is particularly interesting, because it is the outcome of a thorough development process and has been validated across Europe [42, 43] and in other countries around the world [44]. The current version of SWB32 was published in 2019 [44]. As such, it is expected to contribute to a better understanding of the relationship between SWB and QOL [45]. Initially, SWB32 was tested in cancer patients both in palliative and curative care settings [25, 46, 47].

The SWB32 tool addresses patients’ spiritual concerns and can also be seen as an intervention because it prompts reflection on the items it contains, and so gives palliative care professionals and patients an opening to discuss issues in situations where it would otherwise be hard to initiate a conversation [42]. In addition, SWB32 can be useful for detecting patients´ unmet needs, by indicating which aspects of wellbeing are lacking.

Although SWB32 was initially validated with people receiving palliative care for cancer, its use is not restricted to this population [44]. However, large-scale studies are lacking which show the suitability of the SWB32 in patients without cancer. Palliative care is not restricted to cancer patients, and this practical assessment instrument should be available for all patients regardless of their disease.

EORTC QLQ-SWB32 has notable advantages: over 60% of patients find it relatively easy to use and complete in a reasonable time frame [40], a practical guide has been published for its use, and it is available from a non-profit organization at no cost for clinical use. Finally, complete validations in several languages facilitate the use of SWB32 in multicultural comparative studies. Recently, in Nordic countries, SWB32 has been validated in Iceland [48].

So far, metric tools for evaluating spiritual well-being are lacking in our country, yet spirituality is recognized as an important part of palliative and end-of-life care.

## Methods

### Aim

The aim of this study was to produce a Finnish translation of the EORTC QLQ-SWB32, to study its validity and reliability, and its correlation with QOL, among individuals with cancer or other incurable chronic disease either in palliative care or eligible for early palliative care. In addition, associations between the SWB32 health profile and demographic factors were explored.

### Study design for validation

The Finnish translation was produced, pre-tested, validated and re-tested in a prospective manner.

### Translation procedure

The translation from English to Finnish was performed according to the EORTC translation procedure [49]. In summary, two independent translators produced the first Finnish translation, which was harmonized by the research group after consulting three other professional linguists. Thereafter a back-translation to English was obtained from two other independent translators and harmonized by the group. The translation process was carried out by several meetings between the translators over four months. All translators were fluent in English and Finnish, they had been living in English-speaking countries or spoke both Finnish and English as their mother tongue and were familiar with palliative care. The forward and backward translations were submitted to the EORTC language office, which finally approved the Finnish translation for pilot testing.

### Face validity

Face validity for translation was performed on 11 patients in a senior citizen facility in Helsinki. All of the patients were personally interviewed before and after they had filled the questionnaire.

### Setting and participants in the final validation phase

The participants for the validation phase included cancer and non-cancer patients. Cancer patients were recruited by nurses and physicians in Kauniala Hospital, in the outpatient clinics of oncology and palliative medicine at Oulu and Kuopio University Hospitals, Mikkeli and Joensuu Central Hospitals/Palliative care units and through local associations or via advertisements. Non-cancer patients were invited to participate by the researchers, pastors and responsible persons in Christian and Muslim congregations, and non-religious organisations in Finland.

Inclusion criteria for all participants were capacity to fully understand, speak and read Finnish. The cancer patients needed either a defined palliative care plan or the eligibility to receive it. The non-cancer participants needed to be over 65 years and have incurable chronic disease, with a duration of over one year and a continuous need for medication or other care, or severe psychological fear of life-limiting disease. Chronic incurable disease and advanced age together would indicate that these individuals might benefit from early holistic palliative care [50].

The non-cancer individuals were recruited from religious communities because our hypothesis was, that among this group the SWB would be higher than in the cancer group. If this proved the case, this group would represent the positive control group for testing the Finnish translation.

Along with the questionnaire, each patient was given written information concerning the background of the study, explanation of the concept of spirituality and an information sheet with the conditions of participation in the study, ensuring data protection and the possibility of contacting the members of the research team by e-mail or telephone for discussion. Oral and written informed consent was obtained from each of the participants. The health care professionals and volunteers were instructed to adequately communicate the content of the questionnaires and be present and available for discussion at the time of filling it out. This ensured the possibility of dialogue, exchange of impressions and the clarification of possible doubts. After discussing their responses, the patients themselves, the attending staff in the various hospitals, the researcher, or the pastor in non-hospital settings, returned the filled questionnaires in sealed envelopes. They were then stored in a secure place.

### Scoring of the SWB32 scales

SWB32 consists of 31 questions that use a four-point Likert scale, ranging from “Not at all (1)—A little (2)—Quite a bit (3)—Very much (4)” and a 7-point global spiritual well-being (G-SWB) scale from 0= “do not know or cannot answer”, 1= “very poor” to 7= “excellent”. From the primary scales we calculated scores for the following categories as described previously [51]: (1) Relationships with others (RO) (six items), (2) Relationships with self (RS) (five items), (3) Relationship with someone or something greater (RSG) (five items), and (4) Existential issues (EX) (six items). SWB32 also includes a single-item scale: item 26 (RG: Relationship with God). Items 22 and 23 identify patients with a belief for whom the single item scale RG is applicable. The primary validation paper included a fifth category, Change (CH) (four items). These items comprised two for all respondents, and they addressed changes in feelings about life and two for believers only, which addressed changes in beliefs. Such changes could be either positive or negative. A scale score for just these four items is not meaningful. However, they were retained in the measure because they enabled the collection of clinically important information [43].

### Quality of life measurements

In the group of cancer patients, QOL was assessed with Finnish versions of EORTC QLQ-C30 (QLQ-C30) which includes both multi-item scales and single-item scales. All items employ a four-point Likert scale, ranging from 1 (Not at all) to 4 (Very much), except the two 7-point global scales, with a score ranging from 0 to 100. A high score for a functional scale and for the global health status/QOL represents a high/healthy level of functioning and high QOL, while a high score for a symptom scale/item represents a high level of symptomatology/problems [52].

In the non-cancer group, QOL was measured with the Finnish questionnaire − 15D-, which is a generic, 15-dimensional, standardized, self-administered measure of health-related QOL that can be used as a profile and single index score measure (with a range from 1 to 5) in 15 items [53]. The 15D has been developed and widely used in the assessment of QOL among different patient groups in Finland [54, 55].

### Additional information

Information about age, gender, concomitant disease, spread of cancer, treatments, recent hospitalization, living area and membership in any religious communities were collected separately from the main questionnaire. Charlson Comorbidity Index was calculated for each participant using a free software [56].

### Content validity

Five palliative care professionals in Eastern Finland Healthcare District, evaluated the clarity of the meaning of the questionnaire from a health-care practitioners´ view [57].

### Construct validity

Quantitative analysis was carried out for descriptive statistics and the ranges were checked in responses for all items, i.e., where any two response categories account for more than 95% of all responses or any single category less than 5% of responses. Confirmatory factor analysis that utilized principal axis factoring (PAF) and oblique rotation was used for the unified data of all responses. Although the number of factors was based primarily on theory confirmatory graph analysis [58], parallel analysis was also used to assess the optimal number of underlying factors. Model fit was also assessed using traditional measures such as the Tucker-Lewis Index (TLI) and Root-mean-square error of approximation (RMSEA). Internal consistency was assessed using Cronbach’s alpha coefficient [59].

Our hypothesis was that the Finnish translation of SWB32 would appear equally valid and reliable as the original English one and other translations.

### Convergent/divergent validity

RO, RS, RSG, EX, G-SWB and the separate item 26 (RG) were tested for correlations with age, gender, disease-group (cancer/non-cancer), Charlson Comorbidity Index, current hospitalization (hospital) and belonging to religious community, and geographical location, as well as with QLQ-C30 and 15D scores. The scores in the Finnish translation of SWB32 scales were compared with those in the previous non-English translations [25, 46, 47]. We hypothesized that the items in the Finnish translation would have higher scores among individuals connected to religious or other spiritual groups and that the Finnish translation would be psychometrically comparable with other translation of SWB32.

### Re-test

The Finnish version of EORTC QLQ-SWB32 was retested in a group of 16 patients (8 with cancer and 8 without). Re-testing was done two weeks after the first testing.

### Data collection and statistical analysis

Data collection, anonymization, and digitalization were performed in 2020–2021. Findings were expressed in mean values with standard deviations (SD) or percentages, where appropriate. Pearson´s correlation coefficients, t-test, and Chi-square test for pairwise comparisons were calculated for the data using a free soft-ware (PSPP). A correlation coefficient > 0.5 was considered to indicate strong, 0.3–0.5 moderate, 0.2–0.29 weak, and < 0.2 negligible correlation between the items [60]. Statistical significance was set at alpha level p < 0.05.

## Results

### Translation

A consensus regarding the translation of “spiritual as “*henkinen/hengellinen*” in Finnish was achieved during the process. The Finnish translation includes spirituality linked to transcendent or religious aspects (*hengellinen*) and a secular existential experience of personal reality (*henkinen*).

The final wording of the Finnish translation was kept consistent with the original English questionnaire. During the translation process difficulties were encountered in finding a univocal translation faithful to the original English of items 1 (deal with problems), 5 (felt troubled) and 27 (live on through my words). In the end, consensus was achieved in the Finnish translation that reflected the precise meanings of the original English items.

### Participant characteristics

Preliminary testing of the translation was carried out in a pilot group of six female and five male palliative care patients (mean age 82 ± 7.4 years) and separately, content validity was examined in an interview of two female and three male health care professionals (mean age 59 ± 3.3 years). Only the patient data was included in the factorial analysis.

Test-retest was obtained from 16 patients (mean age 72 ± 5.2 years, 8 cancer and 8 non-cancer patients).

A total of 190 participants were included in the final analyses (Table 1).

**Table 1:** Participant’s characteristics. Numbers (N) of patients with percentages (%) and mean (M) with standard deviation (SD). Statistical comparisons between the groups were performed with Chisquare^a^ or t-test^b^, p < 0.05 indicating significant difference and NS non-significant difference

*Variable*	Cancer group N/M (% or SD)	Non-cancer group N/M (% or SD)	P
Total number patients	101 (53%)	89 (47%)	
*Gender*			NS^a^
Female	54 (53.4%)	61 (68.5%)	
Male	47 (46.6%)	28 (31.5%)	
*Age*			< 0.05^b^
Female	70 (10.5)	74 (6.6)	
Male	73 (7.2)	74 (8)	
*Diseases*			< 0.05^b^
Metastatic cancer	60 (59%)		
Non-metastatic cancer	51 (41%)		
Endocrine pathology	29 (28.7%)	21 (23.6%)	
Cardiac pathology	27 (26.7%)	29 (32.6%)	
Neurological pathology	5 (4.9%)	16 (17.8%)	
Respiratory pathology	5 (4.9%)	11 (12.3%)	
Rheumatic pathology	5 (4.9%)	12 (13.5%)	
No additional pathology	30 (29.7%)		
*Charlson Comorbidity Index*			< 0,05^b^
Metastatic cancer	5.5 (1.8)		
Non-metastatic cancer	8.7 (1.4)		
Non-cancer		4.23 (1)	
*Duration of disease*			< 0,05^a^
0–3 months	7 (6.9%)	1 (1.1%)	
> 3 months but < 6 months	8 (7.9%)	1 (1.1%)	
6–12 months	14 (13.8%)	1 (1.1%)	
> 1 year	72 (71.2%)	86 (96.6%)	
*Current place of care*			< 0,05^a^
At hospital	74 (73.3%)	12 (13.5%)	
At home	27 (26.7%)	77 (86.5%)	
*Religious background*			< 0.05^a^
Non-religious	12 (11.9%)	-	
Lutherans	55 (54.4%)	14 (15.7%)	
Orthodox	14 (13.8%)	42 (47.2%)	
Catholics	13 (12.8%)	26 (29.2%)	
Others	2 (1.9%)	-	
No answer	5 (4.9%)	6 (6.7%)	
*Location*			NS^b^
South-Western	76 (75.2%)	41 (46%)	
Mid-Eastern	17 (16.8%)	43 (48.3%)	
Northern	8 (7.9%)	5 (5.6%)	

Cancer patients had significantly higher (p < 0.05) Charlson Comorbidity Index compared to individuals without cancer. 70.3% of the cancer patients also suffered another underlying pathology. Endocrine and cardiac pathologies were the most frequent in both groups, but neurological, respiratory, and rheumatic diseases were more frequent in the non-cancer group. A majority of individuals in both groups had suffered longer than one year, and 32% in the non-cancer group had been hospitalized 1–3 times during the past year because of their incurable diseases. The participants represented a large area of Finland with different dialects and local cultural features.

We were only able to recruit individuals from Christian communities and were unable to get participants from Muslim and atheistic organizations.

Levels of missing data were low. Two patients left without answering 10 items; 8 patients 4 items and 11 patients 3 items: 24–26.

### Face validity

In the pilot testing the questionnaire was regarded as understandable by participants from Kauniala Hospital. Nobody considered any of the items difficult, confusing, upsetting or having difficult words. In addition, no suggestion for other wordings was made. All patients in this pilot testing were baptized Lutherans but not active members in their congregations.

### Content validity

All interviewed health care professionals evaluated the language as understandable and the content as appropriate in terms of terminology and relevance to palliative care.

### Construct validity and reliability

Traditional fit measures in the confirmatory factorial analysis of the item-responses of the entire population (N = 190) (Table 2) indicated an adequate fit for four to six-factor solution (TLI = 0.91, RMSEA = 0.071). The following factors appeared with high Cronbach alpha values indicating good reliability: EX 0.81, RO 0.84, RS 0.73, RSG 0.82, RG 0.85 and CH 0.81.

**Table 2:** Results of factor analysis using principal axis factoring and oblimin rotation. Factor loading < 0.3 are omitted from (h2 = communality, u2 = uniqueness, compl = complexity). For abbreviations of SWB32 factors see Methods

SWB32	item	RO	RSG	EX	CH	RS	h2	u2	compl
RO: I have felt loved by those who are important to me	9	0.80					0.66	0.34	1.1
RO: I have felt able to trust others	11	0.78					0.59	0.41	1
RO: I have felt that I am valued as a person	13	0.77					0.59	0.41	1
RO: I have felt that I have someone to talk to about my feelings	10	0.74					0.6	0.4	1.2
RO: I have felt able to forgive others for things they have done	12	0.66					0.5	0.5	1.7
EX: I have felt that my life is worthwhile	15	0.61					0.57	0.43	1.3
EX: I have felt that my life is fulfilling	14	0.55					0.57	0.43	1.7
RO: I have felt able to share thoughts about life with people who are close to me	8	0.53		0.41			0.57	0.43	2.1
Non-scoring: I have felt able to forgive myself for things I have done	4	0.32					0.36	0.64	3.3
RG: I believe in God or in someone or something greater than myself	22		0.92				0.84	0.16	1
RG: I feel connected to God or to someone or something greater than myself	26		0.85				0.73	0.27	1
RG: I have always believed in God or in someone or something greater than myself	23		0.83				0.72	0.28	1
RSG: I believe in life after death	30		0.83				0.66	0.34	1.1
RSG: I have felt that it is important that other people pray for me	21		0.81				0.67	0.33	1.2
RSG: I have had time for quietness, prayer or meditation	20		0.46				0.39	0.61	2.2
EX: I have felt able to deal with problems	1			0.81			0.67	0.33	1
EX: I have been able to find things I enjoy doing	3			0.76			0.64	0.36	1
EX: I have felt able to plan for the future	16			0.63			0.49	0.51	1.1
RSG: I feel that I will live on through my words, deeds and/or influence on other people	27			0.51			0.27	0.73	1.6
EX: I have felt at peace with myself	2			0.48			0.55	0.45	2.2
CH: My beliefs have changed in the last few weeks	25				0.85		0.69	0.31	1
CH: My beliefs have changed since I have felt less well	24				0.84		0.67	0.33	1.1
CH: My feelings about life have changed in the last few weeks	29				0.65		0.65	0.35	1.4
CH: My feelings about life have changed since I have felt less well	28				0.64	0.33	0.62	0.38	1.5
RS: I have felt that it is unfair that I am ill	19					0.41	0.48	0.52	3.3
RS: I have felt troubled	5					0.8	0.63	0.37	1
RS: I have had worries and/or concerns about the future	17					0.75	0.63	0.37	1.1
Non-scoring: I have worried about the future of people who are important to me	7					0.68	0.43	0.57	1.2
RS: I have felt lonely	6					0.5	0.42	0.58	1.5
RSG: I have spiritual wellbeing	31		0.3			-0.34	0.59	0.41	3.8
RS: I have wondered whether anything can be done for me	18				0.32	0.32	0.38	0.62	3.2

**Fig. 1:**
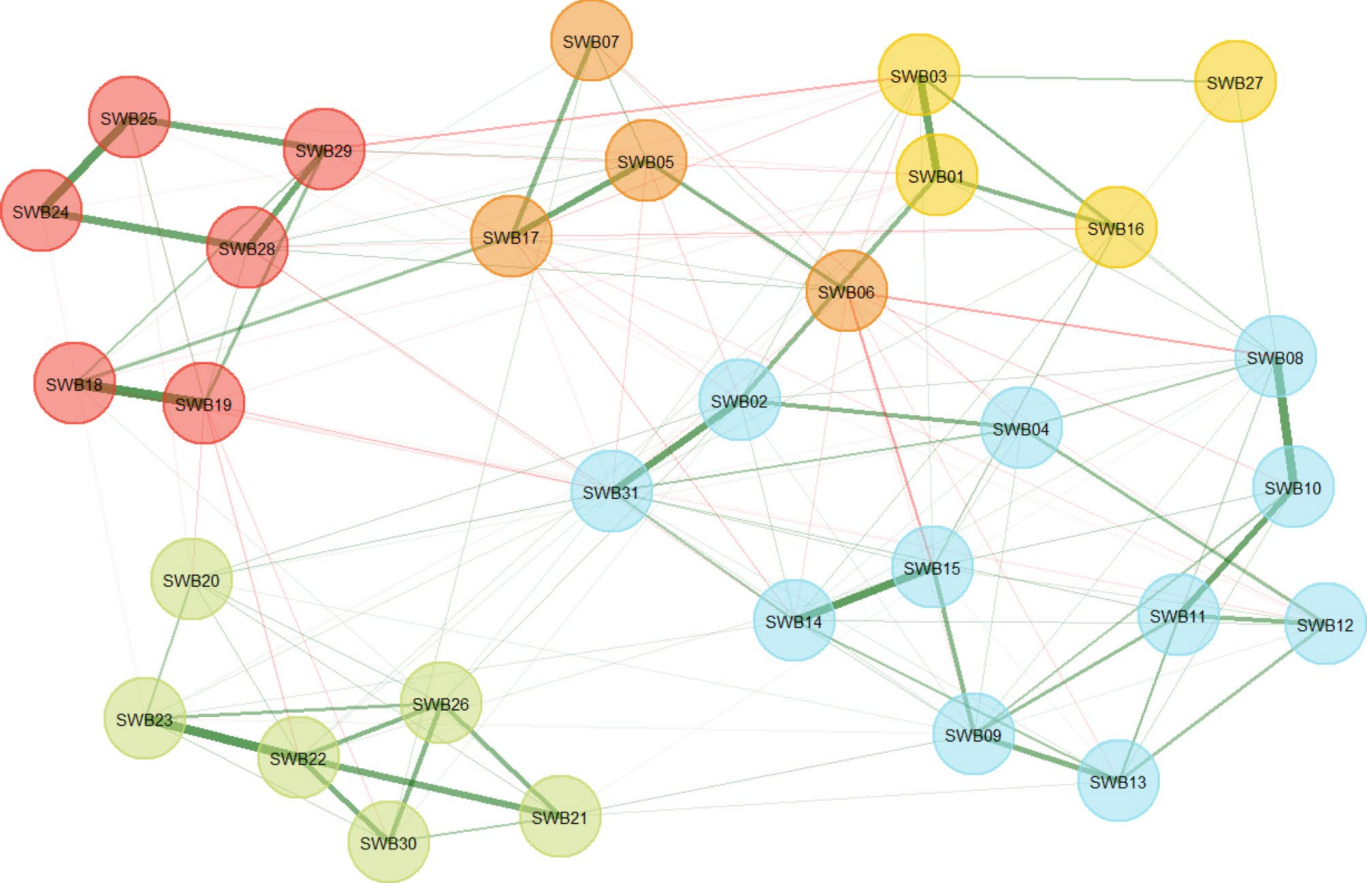
Graph analysis of five factors of SWB32. Coloured areas: *Red*: non-scoring/RS, *Orange*: RS, *Yellow*: EX, *Blue*: EX/RO, and *Green*: RSG/RG. For abbreviations of SWB32 factors see Methods

The connectedness of the factors is illustrated as a graph analysis in the Fig. 1, which shows psychometric overlap of the factors. The mean (SD) absolute values of factors are given in Table 3.

**Table 3:** Mean and standard deviation (SD) values of SWB32 multi-item scales and G-SWB score of all participants and their correlations with age, gender, disease-group (cancer/non-cancer), Charlson Comorbidity Index, current hospitalization (hospital) and religious community. Statistically significant (= p < 0.05) correlations are marked with *. For abbreviations of SWB32 factors see Methods

Mean (SD)	RS	RSG	EX	RG	G-SWB	Age	Gender	Disease	Charlson	Hospital	Religious community
RO 78.5 (16.8)	0.308*	0.364*	0.528*	0.187*	0.223*	0.047	0.039	0.04	0.003	-0.031	0.022
RS 64.7 (19.9)		0.286*	0.523*	0.134	0.390*	0.092	0.166	0.17	-0.190	-0.197	0.308*
RSG 72.2 (18.1)			0.455*	0.607*	0.506*	0.202*	0.287*	0.287*	-0.182	-0.373*	0.487*
EX 75.7 (18.0)				0.267*	0.573*	0.048	0.202*	0.202*	-0.180	-0.249	0.174
RG 67.7 (40.3)					0.411*	0.029	0.229*	0.229*	-0.187	-0.366*	0.393*
G-SWB 67.9 (27.2)						0.061	0.223*	0.223*	-0.181	-0.301*	0.327*

### Convergent/divergent validity

*Health-related and demographic issues*. SWB32 measures did not correlate with Charlson Comorbidity Index (Table 3). We found no connection between any component of SWB32 and the geographical living area of the participant. However, G-SWB correlated positively (p < 0.05) with female gender and belonging to a religious community. These correlations were stronger than those for disease-related ones. A negative correlation was observed between RSG, RG and G-SWB and hospitalisation.

*Quality of life*. Completed QOL questionnaires were obtained from 74 cancer (QLQ-C30) and 72 non-cancer (15D) patients. RS, RSG, EX, RG and G-SWB were significantly higher in the non-cancer group compared to the cancer group. The correlations between multi-item scales, RG and G-SWB score with QLQ-C30 in the cancer group are presented in Table 4. A significant (p < 0.05) positive correlation was found between G-SWB and global health status and emotional functioning, while there was a negative correlation between G-SWB and dyspnea, insomnia and financial difficulties.

**Table 4:** Cancer group. Mean and standard deviation (SD) values of QLQ-C30 scores and their correlations with multi-item scales, RG and G-SWB score of SWB32. Statistically significant (= p < 0.05) correlations are marked with *. For abbreviations of SWB32 factors see Methods. QL2: Global health status/QOL; PF2: physical; RF2: role; EF: emotional; CF: cognitive; SF: social; FA: fatigue; NV: nausea and vomiting; PA: pain; DY: dyspnea; SL: insomnia; AP: appetite loss; CO: constipation; DI: diarrhea; FI: financial difficulties

QLQ-C30 / SWB32	QL2	PF2	RF2	EF	CF	SF	FA	NV	PA	DY	SL	AP	CO	DI	FI
Mean (SD)	60.1 (23.4)	59 (27.7)	63.6 (33.3)	79.2 (18.5)	82.0 (23.1)	74.9 (23.1)	43,2 (26.1)	11,7 (19.3)	30.1 (31.9)	26 (30.9)	30.3 (28.2)	24.2 (35.2)	17.8 (26.8)	13.6 (23.2)	19.2 (32.6)
RO 77.9 (17.2)	0.098	-0.221*	-0.098	0.317*	0.070	-0.030	0.014	0.139	0.027	-0.187	-0.165	0.185	0.66	0.11	-0.086
RS 61.7 (20.7)	0.179	0.152	0.228*	0.591*	0.117	0.219*	-0.234*	-0.004	-0.170	-0.244*	-0.300*	0.050	0.004	-0.155	-0.309*
RSG 67.4 (19.1)	0.251*	0.087	0.022	0.265*	0.158	0.101	-0.123	0.004	0.065	-0.212*	-0.095	-0.017	0.112	0.004	-0.165
EX 73.4 (17.5)	0.482*	0.148	0.312*	0.557*	0.272*	0.291*	-0.313*	-0.012	-0.039	-0.396*	-0.205	-0.113	-0.150	-0.097	-0.254
RG 59.1 (42.7)	0.190	0.048	0.082	0.190	0.107	-0.38	-0.122	-0.68	-0.20	-0.194	-0.040	-0.122	-0.011	-0.175	-0.113
G-SWB 62.2 (30.4)	0.345*	0.056	0.132	0.255*	0.107	0.113	-0.193	-0.044	-0.97	-0.38*	-0.209*	-0.057	-0.081	-0.106	-0.333*

In the non-cancer group, there were multiple positive significant correlations between multi-item scales, RG and G-SWB score with both the 15D index and individual 15D scores (Table 5). The G-SWB, RS and EX were significantly associated (p < 0.05) with the 15D index of QOL. RS and EX are also significantly correlated to the 15D index. Significant negative correlations were not detected in this group.

**Table 5:** Non-cancer group. Mean and standard deviation (SD) values of 15D scores and their correlations with multi-item scales, RG and G-SWB score of SWB32. Statistically significant (= p < 0.05) correlations are marked with *. For abbreviations of SWB32 factors see Methods. Mov: mobility; Vis: vision; Hea: hearing; Bre: breathing; Sle: sleeping; Eat: eating; Spe: speech; Eli: elimination; Act: usual activities; Men: mental function; Disc: discomfort, Dep: depression; Dist: distress; Vit: vitality; Sxa: sexual activity. G-SWB: global spiritual well-being q.32

15D / SWB32	15D Index	Mov	Vis	Hea	Bre	Sle	Eat	Spe	Eli	Act	Men	Disc	Dep	Dist	Vit	Sxa
Mean (SD)	0.84 (0.13)	0.80 (0.26)	0.91 (0.17)	0.92 (0.14)	0.82 (0.24)	0.77 (0.20)	0.96 (0.14)	0.94 (0.15)	0.78 (0.25)	0.76 (0.29)	0.84 (0.22)	0.68 (0.21)	0.87 (0.16)	0.86 (0.17)	0.82 (0.19)	0.81 (0.29)
RO 79.2 (15.5)	0.186	-0.037	-0.078	0.093	0.076	0.207*	-0.087	0.13	0.208*	-0.053	0.214*	0.119	0.351*	0.234*	0.257*	0.131
RS 68.3 (18.4)	0.358*	0.197	0.197	-0.037	-0.13	0.308*	0.014	-0.112	0.157	0.251*	0.073	0.246*	0.603*	0.528*	0.309*	0.358*
RSG 77.6 (15.1)	0.184	0.092	0.0	-0.038	-0.18	0.056	0.133	0.064	0.247*	0.045	0.152	0.071	0.240*	0.326*	0.209*	0.247*
EX 79.6 (17.9)	0.668*	0.437	0.283*	0.149	0.157	0.276	0.280*	0.252*	0.364*	0.462*	0.403*	0.393*	0.754*	0.609*	0.635*	0.441*
RG 77.5 (35.1)	0.136	0.06	-0.021	-0.079	-0.141	0.124	0.072	0.065	0.271*	-0.028	0.128	0.37*	0.108	0.254*	0.200*	0.151
G-SWB 74.3 (21.6)	0.419*	0.283	0.068	-0.002	-0.063	0.109	0.087	0.241	0.331*	0.207*	0.342*	0.203*	0.469*	0.565*	0.379*	0.437*

Finally, the comparison of scores in the Finnish translation of SWB32 scales with other translations is presented in Table 6.

**Table 6:** Comparison of the mean and standard deviation (SD) scores of the SWB32 scales in this study (Finland) and previous studies. NA = not available

	*China* Chen et al. 2021^46^	*Cyprus* Kyranou et al. 2021^47^	*Croatia* Dabo et al. 2021^25^	*Finland* *Current study* 2022
Participants	705	104	143	190
Relationship with Others (RO)	70.69 (13)	82.3 (18.9)	72.22 (19.4)	78.5 (16.8)
Relationships with Self (RS)	75.22 (11)	45.2 (23.7)	73.33 (20.0)	64.7 (19.9)
Relationship with Someone or Something Greater (RSG)	52.2 (11.8)	64.6 (22.0)	60 (23.3)	72.2 (18.1)
Existential (Ex)	68.4 (13.3)	69.7 (22.0)	72.22 (26.61)	75.7 (18.0)
Relationship with God (RG)	NA	74.9 (29.7)	33.33 (25.0)	67.7 (40.3)
G-SWB	72.48 (35.0)	60.4 (28.7)	66.67 (20.8)	67.9 (27.2)

### Re-test reliability

There were no statistically significant differences between the first and second responses.

## Discussion

### Main findings

We tested and validated a Finnish translation of the SWB32 questionnaire which showed a high reliability. In addition, we have shown that G-SWB correlates with QOL as measured by both QLQ-C30 and 15D for individuals eligible for palliative care with or without cancer.

In English, the word “spirituality” has a wide range of meaning that does not fully translate into Finnish. In modern academic Finnish “spiritual” is translated as “*spirituaalinen*” [61]. Since non-academics might not understand this meaning, this modern academic translation was not used. Instead, following the conclusions from other Finnish studies [62], the double expression “*henkinen/hengellinen*” which corresponds to the broader sense of “spirituality” in English was used.

In the Finnish translation the individual items of SWB32 were successfully loaded in four to six factors. The overall structure of the SWB questionnaire in Finnish was similar to the original [44]. Good factorial loading, high Cronbach alfa values and a minimal loss of data indicate that the Finnish translation is valid and reliable. The previously suggested factors RO, RS, EX, RSG, the single item RG, and G-SWB, are useful practical categories in the Finnish version of EORTC-SWB32 as well [48]. The non-scoring items (4,7) of the original translation had the least factorial loading in Finnish as previously reported [44] but are recommended as additional items to facilitate discussions about spiritual experiences and patient needs. Because a four factor -loading with exclusion of change (CH) factor also appeared possible, we did not explore relationships between CH and other observations. According to Vivat et al. (2017) [44], change may not be a clinically relevant category, because its values may vary inconsistently from negative to positive. Items in the original CH factor are considered non-scoring items that are useful for comprehensive assessment of SWB.

Absolute mean values of most subcategories of SWB32 and G-SWB of cancer patients in our studies are similar to those in previous studies [25, 46, 47]. We concluded that this observation underscores the high validity of our Finnish translation. In the absence of cut-off values, the comparison of translations requires further studies.

In the Finnish spoken language, the term “spiritual” may be confused with “religious”, which we wanted to avoid. This is why the concepts of spirituality and religion were clarified to the participants before they filled the questionnaires using a national consensus on terminology in palliative care. Good spiritual well-being could be experienced without religious connections [14].

### Spiritual well-being and quality of life

We chose two measures of quality of life, QLQ-C30 and 15D, which have often been used in Finnish studies concerning QOL in cancer and non-cancer patients [63–65]. The mean global QOL scores (60/100 in QLQ-C30 and 0.8/1 in 15D) indicate that the QOL was good among the participants in the current study.

In accordance with previous studies [31, 47, 66, 67], we observed that in QLQ-C30, the emotional functioning scales were positively correlated with spirituality but symptom scales for dyspnoea, fatigue and insomnia were negatively correlated with various scales in SWB32. For instance, in our study, fatigue was the most frequent single symptom with a negative correlation with RS and EX. We also detected similar correlations with 15D which have not been previously studied in conjunction with spiritual issues. Special attention should therefore be paid to the spiritual issues of physically debilitated and suffering patients. In support of this, Brandão et al. [68] suggest that high levels of spirituality in patients may lead to better endurance of physical symptoms. Spirituality has been recognized by researchers, clinicians, and patients as an important resource for addressing distress when facing death [69, 70]. We however agree with Garssen and Visser that caution is needed when associating spirituality with the prediction of distress or depression [71].

Further studies are needed to determine whether the use of SWB32 might detect patient spiritual suffering as manifested in physical symptoms, or unmet spiritual needs in palliative care [31].

### Spirituality, morbidity, and demographic factors

In the current study, SWB32 scores were higher in the non-cancer group compared to the cancer group. We hypothesise that this reflects the differences in religious affiliations between the groups because SWB32 did not correlate with Charlson Comorbidity Index. There were significant positive correlations between SWB32 and belonging to religious groups among all participants. The number of individuals in different Christian confessions was too small in both study groups to allow any conclusions regarding their role in the participants´ SWB and QOL.

Female gender was associated with higher scores in SWB32 which agrees with previous studies [25, 31, 72]. However, areas with different Finnish dialects did not have any measurable influence on SWB32. We did not conduct differential item functioning (DIF) analyses for age because most of the patients were in the same age bracket. Further studies on SWB could investigate the difference between hospital and home-based palliative care.

### Strengths and limitations

The population of 190 patients in this study assessing the validity and reliability of SWB32 is the largest from a single cultural environment in Europe. Previous validation studies have included only 7 to 143 individuals [25, 44, 47, 48]. The participants were drawn from 11 cities in the three largest areas of Finland, representing the different idiosyncrasies and dialects in our country. In addition, missing data was very low in our study.

Another merit of our study is that we executed the validation of SWB32 in patients with and without cancer. Our study is one of the few studies which has assessed spiritual issues in non-cancer patients and in early palliative care [67, 73]. Finally, our study is the first one, to the best of our knowledge, to study the interaction between 15D and SWB32.

One of our primary goals was to test the Finnish translation with patients belonging to spiritual communities. We assumed that spirituality and SWB would be important for them, and their scores in SWB would be high. Unfortunately, we were unable to recruit patients from non-Christian spiritual organizations.

We invited participation from three Muslim organizations but they answered that participation would be impossible due to the lack of communication in Finnish with the potential patients. A Muslim physician unsuccessfully attempted to recruit participants from that population. In addition, we contacted a representative of an atheistic organization but again without success. It must be noted however, that in Finland 75% of the population belongs to Christian Churches [74]. In our study 81% of the cancer group and 92% of the non-cancer group were Christian. Thus, our sample of individuals represents the national average, and the translation would be suitable for the majority of Finns. Future studies may be required to assess the Finnish translation in individuals from different spiritual backgrounds.

Finally, a limitation of our study was that we did not have access to the medical records of the participants but only relied on the patients´ personal report of their medical history. A more robust understanding of the relation between disease and SWB could be achieved with access to accurate information concerning the medical condition of the patients.

## Conclusions

We have provided a Finnish translation of the EORTC QLQ-SWB32 questionnaire and demonstrated its high validity and reliability among patients in palliative care or eligible for it. EORTC QLQ-SWB32 could serve as a useful tool in clinical care and research for assessment of spiritual and existential issues. A positive correlation was found between SWB, QOL and belonging to a religious community.

## Data Availability

The raw data collected during the current study is not publicly available due to state restrictions as it contains information that could compromise research participant privacy/consent but are available from the corresponding author on reasonable request.

## List of abbreviations

EORTC: European Organisation for Research and Treatment of Cancer
QLQ-SWB32: Quality of Life Questionnaire-Spiritual Well-Being 32
QOL: Quality of Life
QLQ-C30: Quality of Life Questionnaire-C30
15D: Quality of Life Questionnaire-15D
RO: Relationships with others
RS: Relationships with self
RSG: Relationship with someone or something greater
EX: Existential issues
CH: Change
G-SWB: Global spiritual well-being
QL2: Global health status/Quality of Life
PF2: Physical
RF2: Role
EF: Emotional
CF: Cognitive
SF: Social
FA: Fatigue
NV: Nausea and vomiting
PA: Pain
DY: Dyspnea
SL: Insomnia
AP: Appetite loss
CO: Constipation
DI: Diarrhea
FI: Financial difficulties
Mov: Mobility
Vis: Vision
Hea: Hearing
Bre: Breathing
Sle: Sleeping
Eat: Eating
Spe: Speech
Eli: Elimination
Act: Usual activities
Men: Mental function
Disc: Discomfort
Dep: Depression
Dist: Distress
Vit: Vitality
Sxa: Sexual activity
PAF: Principal axis factoring
TLI: Tucker-Lewis Index
RMSEA: Root-mean-square error of approximation
